# Early effects of lurasidone treatment in a chronic mild stress model in male rats

**DOI:** 10.1007/s00213-023-06343-5

**Published:** 2023-02-23

**Authors:** Kerstin Camile Creutzberg, Veronica Begni, Francesca Marchisella, Mariusz Papp, Marco Andrea Riva

**Affiliations:** 1 Department of Pharmacological and Biomolecular Sciences, University of Milan, Via Balzaretti 9, 20133 Milan, Italy; 2 Biological Psychiatry Unit, IRCCS Istituto Centro San Giovanni di Dio Fatebenefratelli, Via Pilastroni 4, 25125 Brescia, Italy; 3 Maj Institute of Pharmacology, Polish Academy of Sciences, Smętna 12, 31-343 Krakow, Poland

**Keywords:** Treatment responsiveness, Chronic mild stress, Prefrontal cortex, Activity-dependent genes, Excitatory inhibitory balance

## Abstract

**Rationale:**

Stress represents a major contributor to the development of mental illness. Accordingly, exposure of adult rats to chronic stress represents a valuable tool to investigate the ability of a pharmacological intervention to counteract the adverse effects produced by stress exposure.

**Objectives:**

The aim of this study was to perform a time course analysis of the treatment with the antipsychotic drug lurasidone in normalizing the anhedonic phenotype in the chronic mild stress (CMS) model in order to identify early mechanisms that may contribute to its therapeutic activity.

**Methods:**

Male Wistar rats were exposed to CMS or left undisturbed for 7 weeks. After two weeks of stress, both controls and CMS rats were randomly divided into two subgroups that received vehicle or lurasidone for five weeks. Weekly measures of sucrose intake were recorded to evaluate anhedonic behavior, and animals were sacrificed at different weeks of treatment for molecular analyses.

**Results:**

We found that CMS-induced anhedonia was progressively improved by lurasidone treatment. Interestingly, after two weeks of lurasidone treatment, 50% of the animals showed a full recovery of the phenotype, which was associated with increased activation of the prefrontal and recruitment of parvalbumin-positive cells that may lead to a restoration of excitatory/inhibitory balance.

**Conclusion:**

These results suggest that the capacity of lurasidone to normalize anhedonia at an early stage of treatment may depend on its ability to modulate the function of the prefrontal cortex.

**Supplementary Information:**

The online version contains supplementary material available at 10.1007/s00213-023-06343-5.

## Introduction

Mental illnesses are complex diseases comprising significant alterations of different behavioral domains. Anhedonia, defined as a decreased capacity to feel pleasure, is a key symptom of such disorders, including schizophrenia and depression, and represents an important target for therapeutic intervention aimed at improving the quality of life of affected individuals. Together with the genetic background, stress exposure represents a major risk factor for the development of such conditions (Park et al. 2019). Accordingly, the investigation of the functional and molecular changes produced by stress exposure in animal models can be instrumental to increase the knowledge of the brain mechanisms that may be targeted by therapeutic intervention. The chronic mild stress (CMS) paradigm is an established protocol that is able to generate depressive-like behavior in rodents (Willner 2005), including a decreased preference for the intake of sweet solutions resembling an anhedonic phenotype, as well as cognitive deficits. A range of drugs has been tested and proven to be effective in reversing CMS-induced deficits after chronic treatment (Muñoz & Papp 1999; Paladini et al. 2021; Rossetti et al. 2016; Willner et al. 1987). We have recently demonstrated that the antipsychotic drug lurasidone can normalize the anhedonic phenotype in animals exposed to CMS (Begni et al. 2022; Brivio et al. 2021; Calabrese et al. 2020; Calabrese et al. 2016; Luoni et al. 2014; Tarazi & Riva 2013), a result that is in line with the clinical evidence demonstrating the ability of lurasidone to improve depressive symptomatology in patients (Loebel et al. 2014; Wolke et al. 2019).

As for other drugs, the effect of lurasidone develops progressively and reaches a complete normalization of the anhedonic phenotype by the 4th–5th week of treatment (Begni et al. 2022; Brivio et al. 2021; Calabrese et al. 2020; Calabrese et al. 2016; Luoni et al. 2014). Interestingly, we have identified different CMS-induced molecular changes that were modulated by lurasidone at the end of treatment. Such changes could be part of the mechanisms that contribute to the normalization of the pathologic phenotype but could also represent an adaptive consequence of the functional improvement following lurasidone administration in CMS rats. However, little is known about brain changes during the early phase of the treatment that can be predictive of drug responsiveness and may represent an important element to better characterize the therapeutic properties of a given molecule. With that in mind, the aim of this study was to perform a time course analysis of the changes produced by the antipsychotic drug lurasidone in the CMS model in order to identify rats that may show an early response to the treatment and characterize the molecular alterations that may trigger the improvement of the anhedonic phenotype.

## Methods

### Experimental design and animal housing

Male Wistar rats were purchased from Charles River (Germany) and were delivered to the animal facility one month before the beginning of the experiment. Animals were randomly divided into two groups, control (CT) and chronic mild stress (CMS), each group with an *n* of 80 animals. The experiment had a total duration of seven weeks, and sucrose intake was monitored weekly. During the first two weeks, CMS animals were exposed to the stress protocol while CT animals were left undisturbed, except for cage cleaning and sucrose measurements. From week three, each group was randomized to receive either vehicle or lurasidone, resulting in four subgroups of 40 animals, CT treated with vehicle (CT/VEH), CT treated with lurasidone (CT/LUR), CMS treated with vehicle (CMS/VEH), and CMS treated with lurasidone (CMS/LUR). At the end of weeks 3, 4, 5, and 7, a batch of each subgroup of animals (*n* = 10 per group) was sacrificed.

Animals were single-housed and had access to food and water ad libitum, except during food and water deprivation of CMS protocol and sucrose intake measurements. Moreover, cages were maintained on a 12-h light/dark cycle (lights on at 08 am) in an environment with controlled temperature (22 ± 20 °C) and humidity (50 ± 5%) conditions. All procedures included in this study comply with the ARRIVE guidelines are in conformity with the rules and principles of the EU Directive 2010/63/EU and have been approved by the Local Bioethical Committee at the Institute of Pharmacology, Polish Academy of Sciences, Krakow, Poland.

### Sucrose intake and stress protocol

Animals were trained to consume 1% sucrose solution for one week prior to the beginning of the experiment. The training consisted of eight 1-h baseline tests, in which sucrose was presented, in the home cage, following 14 h of food and water deprivation. Sucrose bottles were weighed and placed in the home cage for one hour; after this period, bottles were re-weighed and sucrose intake was calculated. Subsequently, sucrose intake was measured weekly throughout the whole experiment. Animals were given only one bottle (containing the sucrose solution) during the test.

Animals allocated to the CMS group were exposed to a chronic mild stress protocol for a period of up to seven consecutive weeks. Each week of the stress protocol was composed by the following: two periods of food or water deprivation, two periods of 45-degree cage tilt, two periods of intermittent illumination (lights on and off every 2h), two periods of a soiled cage (250 ml water in sawdust bedding), one period of paired housing, two periods of low-intensity stroboscopic illumination (150 flashes/min), and three periods of no stress. Every period had a duration of 10 to 14 hours without interruptions (day and night), and the sequence of stressors was different every week to avoid habituation. Control animals were housed in separate rooms and had no contact with stressed animals.

### Drug administration

After two weeks of stress exposure, CT and CMS animals were further divided into matched subgroups, considering their sucrose intake. For the next five weeks, animals received one oral daily administration of vehicle (1% (w/v) hydroxy-ethylcellulose) or lurasidone (3.0 mg/kg) per gavage. The volume of all inoculations was set at 1 ml/kg. Moreover, drug administration occurred around 10 am, and the sucrose intake test was carried out at weekly intervals, 24 h after drug administration.

### Sacrifice and biological sampling

Twenty-four hours after one, two, three, and five weeks of drug administration, 10 animals from each group were decapitated and brain samples were collected. The night before sacrifice, CMS animals received tilting or no stress; otherwise, scheduled stressors were applied. Animals were individually removed from their housing rooms in a semi-randomized order.

After sacrifice, the brain was extracted from the skull and placed on an ice-chilled plate. Brains were cut in half to divide the two hemispheres. The left hemisphere was free-hand dissected in the same ice-chilled to collect the following brain regions: the prefrontal cortex (PFC), ventral hippocampus (VH), dorsal hippocampus (DH), amygdala (Amy), and nucleus accumbens (NAc). Dissected tissues were snap-frozen using dry ice and stored at −80 °C until RNA extraction. The right hemisphere was immediately transferred to an ice-cold 50-ml tube pre-filled with 4% paraformaldehyde (PFA). Tubes were kept at 4 °C for at least 24 h (until the brains became whitish). Subsequently, PFA was replaced by a 30% sucrose solution (dissolved in phosphate-buffered saline – PBS 1X), and tubes were kept at 4 °C until the sinking of the brains to the bottom of the tube (~48 hours). Then, brains were snap-frozen in 2-methylbutane, cooled with dry ice, placed in aluminum foil, and stored at −80 °C until further processing.

### RNA extraction and transcriptional analysis

Total RNA was extracted from all brain regions (PFC, VH, DH, Amy, and NAc) from all animals sacrificed after two weeks of lurasidone/vehicle administration. RNA extraction was performed using PureZol RNA isolation reagent (Bio-Rad Laboratories, Italy) standard protocol, following manufacturers’ instructions. RNA concentration was measured with NanoDrop spectrophotometer (ThermoFisher), and one aliquot of each sample was further treated with DNase to avoid DNA contamination. Following DNase treatment, samples were diluted at 10 ng/ul and were used for quantitative real-time polymerase chain reaction (qRT-PCR) (CFX384 real-time system, Bio-Rad Laboratories, Italy). All samples were run in a 384-well plate in triplicate with ß-Actin as an internal control (housekeeping gene). Primers for Arc (Rn00571208_g1), Zif-268 (Rn00561138_m1), and Npas4 (Rn01454622_g1) were purchased from Thermo Fisher Scientific while ß-actin (Fwd: CACTTTCTACAATGAGCTGCG, Rev: CTGGATGGCTACGTACATGG, probe: TCTGGGTCATCTTTTCACGGTTGGC) primer and probe from Eurofins Genomics. The efficiency-corrected model was used for qRT-PCR analysis, in which the amplification efficiencies of target and housekeeping genes were considered (Pfaffl 2001). Data are presented as fold change % compared to the CT/VEH group (set at 100%).

### RNAscope in situ hybridization and immunofluorescence

In accordance with the rat brain atlas (Paxinos & Watson 2006), the whole extension of the PFC of post-fixed frozen brains was cut into 30-µm coronal sections with a microtome (*n* = 4 per group). One out of six sections was mounted on positively charged microscopic glass slides (Thermo Fisher Scientific). Rat-Peptidylprolyl Isomerase B (Rn-Ppib – 313921) was used as a positive control probe and Npas4 as the target RNA probe (Rn-Npas4 – 493881), and Ppib and Npas4 were used in separated brain slices. The protocol, RNAscope™ Fluorescent Multiplex Assay, was followed as indicated by the manufacturer.

Immunofluorescence was performed immediately after the in-situ hybridization protocol, as previously published (Marchisella et al. 2021). The following primary antibodies were used: rabbit anti-parvalbumin (#NB120-11427, Novus Bio) to stain parvalbumin cells (1:1000) and mouse anti-calcium calmodulin kinase II (MA1-048, Thermo Fischer) for pyramidal interneurons (1:100). Detection was obtained with Alexa dye-conjugated antibodies (anti-rabbit 488 and anti-mouse 647, respectively) at a concentration of 1:500. Sections were cover-slipped with fluorescent mounting medium ProLong Gold Antifade reagent (Thermo Fisher Scientific) containing DAPI for nuclei visualization.

Images were acquired with an LSM-900 confocal microscope (Carl Zeiss, Oberkochen, Germany) using a 10× objective to navigate the area of interest and 63× to snap the image, which was further used to analyze the fluorescent signal. Signal detection quantification was performed by an experimenter blind to the experimental group using ImageJ (National Institute of Health) software. Parvalbumin and CAMKII-positive neurons were first identified to be colocalized with DAPI. RNA transcript signals of the gene probes (Ppib or Npas4) appeared as punctate dots in separated brain slices. We considered nuclear staining to account for somatic localization of RNA transcripts/puncta signals, and the total number of puncta in every parvalbumin or CAMKII-positive cell and the average number of puncta per animal was calculated. In the case of puncta overlap, the area of the cluster was divided by the area of 5 different and random puncta in the same field. The total count of Npas4 puncta was normalized using the Ppib count.

### Statistical analysis

Data were analyzed using IBM SPSS Statistics v.27 and GraphPad Prism 8. Differences between groups were analyzed using one-way ANOVA with multiple comparison tests (Tukey’s *post hoc* test). To investigate the alterations in sucrose intake throughout the weeks, we performed a 3-way ANOVA with time, treatment, and stress as factors. Data are presented as group mean ± standard error of the mean (SEM). Outlier analysis was conducted, and samples classified as outliers were removed from the molecular analysis. The graphs represent individuals as dots, and a *p*-value <0.05 was considered statistically significant.

A *Z*-score was calculated considering the immediate early genes (IEGs) that were analyzed with qRT-PCR (*Arc*, *Npas4*, and *Zif-268*) to have an integrated overview of the molecular analysis. The *Z*-activation scores were obtained by averaging the *z*-scores of the % fold change of the IEGS. The *Z*-activation was calculated only when the animal had the qRT-PCR results from all three genes. The individual *z*-score per animal in each test was obtained by subtracting the group average value from the sample value and then dividing it by the group’s SD.

## Results

### Behavioral effect of lurasidone on CMS-induced anhedonia

**Fig. 1:**
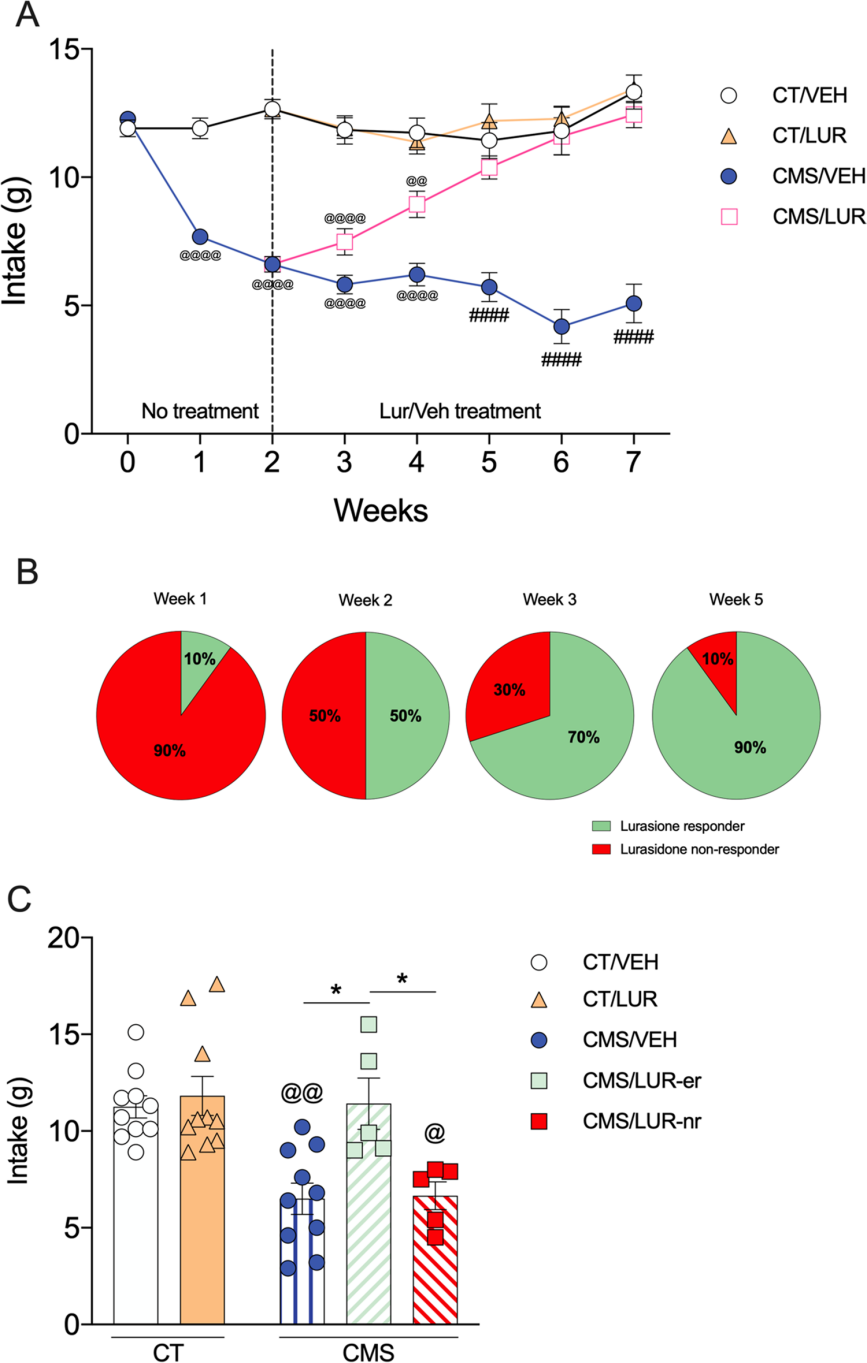
Sucrose intake after CMS exposure and modulation by chronic lurasidone treatment. **A** Sucrose intake was measured at weekly intervals in control (CT) or stressed (CMS) animals treated with vehicle (VEH) or lurasidone (LUR). Statistical analysis: three-way ANOVA, Tukey’s post hoc, ^@@@@^*p* < 0.0001 vs. CT groups (CT/VEH and CT/LUR), ^@@^*p* < 0.01 vs. CT groups (CT/VEH and CT/LUR), ^####^*p* < 0.0001 vs. every other group. Results are expressed as mean ± SEM, *n* = 10 animals per group. **B** Identification of the percentage of CMS animals that were responders or non-responders to lurasidone treatment. **C** Analysis of sucrose intake measured at week 2 of treatment in CT or CMS rats, discriminating responders (CMS/LUR-er) or non-responders (CMS/LUR-nr) to lurasidone treatment. Statistical analysis: one-way ANOVA, ^@@^*p* < 0.01 vs. CT groups (CT/VEH and CT/LUR), ^@^*p* < 0.05 vs. CT groups (CT/VEH and CT/LUR), *p < 0.05. Results are expressed as mean ± SEM, *n* = 5–10 animals per group

The behavioral analysis (3-way ANOVA) revealed significant main effects for time (*F* (7, 882) = 15.7, *p* < 0.0001), stress (*F* (1, 882) = 325.4, *p* < 0.0001), and treatment (*F* (1, 882) = 53.59, *p* < 0.0001). Moreover, we found significant time × stress (*F* (7, 882) = 17.89, *p* < 0.0001), time × treatment (*F* (7, 882) = 5.58, *p* < 0.0001), stress × treatment (*F* (1, 882) = 58.94, *p* < 0.0001), as well as time × stress × treatment (*F* (7, 882) = 3.11, *p* = 0.003) interactions. Tukey’s *post hoc* test revealed that at baseline (week 0), when animals were randomized into two experimental groups, namely control (CT) and chronic mild stress (CMS), no differences were found in sucrose intake. In line with our previous work (Begni et al. 2022; Luoni et al. 2014), exposure to CMS produces an anhedonic phenotype that becomes evident after the first week of stress. Indeed, CMS animals showed a significant reduction in sucrose intake when compared to control animals at weeks 1 and 2 (*p* < 0.0001). Animals were then randomized to receive either vehicle or lurasidone for 5 weeks while continuing stress exposure. As shown in Fig. 1A, CMS rats treated with VEH (CMS/VEH) showed decreased sucrose intake throughout the whole experiment (*p* < 0.0001 for weeks 3 to 7) when compared to CT/VEH group. Conversely, CMS rats treated with lurasidone (CMS/LUR) showed a progressive improvement of the anhedonic phenotype, with a complete recovery starting from the end of the third week of treatment. Accordingly, when compared to CT animals treated with vehicle (CT/VEH), CMS/LUR animals showed a significantly decreased sucrose intake only during the first two weeks of treatment (*p* < 0.0001 for week 3; *p* = 0.0043 for week 4), while no significant differences were found during the final weeks of the experiment (weeks 5 to 7). Moreover, the CT/VEH and CT/LUR groups did not show significant differences at any time point.

The main goal of this study was to identify among CMS rats that received lurasidone, those that may be considered early treatment responders. In order to do so, CMS/LUR animals were classified as responders or non-responders to lurasidone treatment. To be considered a responder, the sucrose intake value of the animal needed to be higher or equal to the sum of the average sucrose intake of the CT/VEH group and one standard deviation (SD) (sucrose intake > average sucrose intake CT/VEH group + 1SD sucrose intake CT/VEH group) (Ardi et al. 2016). If the animal responded to the treatment in the first or second week of drug administration, it was considered an early responder (CMS/LUR-er), while the others were considered non-responders (CMS/LUR-nr). Based on this categorization, we found that at week 1, 10% (*n* = 1) of CMS/LUR animals could be considered responders to lurasidone, a percentage that increased to 50% (*n* = 5) in the second week, 70% (*n* = 7) at the end of the third week, and to 90% (*n* = 9) at the last time point (fifth week of treatment) (Fig. 1B).

On these bases, we decided to focus on the second week of treatment, when we were able to identify an equal number of animals within the two subgroups of CMS rats treated with lurasidone: the CMS/LUR-er (“early responders”) that show a complete recovery from the anhedonic phenotype (vs. CT/VEH; Fig. 1C) and the CMS/LUR-nr (“non-responders”), whose sucrose intake was still significantly reduced when compared to CT/VEH (*p* = 0.0175), and that was also statistically different from CMS/LUR-er (*p* = 0.0402; Fig. 1C).

### Analysis of activity-regulated genes in early vs. late responders to lurasidone

Based on the behavioral data, we focused on the second week of treatment to identify molecular changes that may contribute to the early responsiveness to lurasidone. Hence, we investigated the expression of three genes, namely *Arc*, *Zif-268*, and *Npas4*, which respond to different signaling pathways and are considered good markers for the activity state of a given brain region (Fu et al. 2020; Korb & Finkbeiner 2011; Veyrac et al. 2014).

**Fig. 2:**
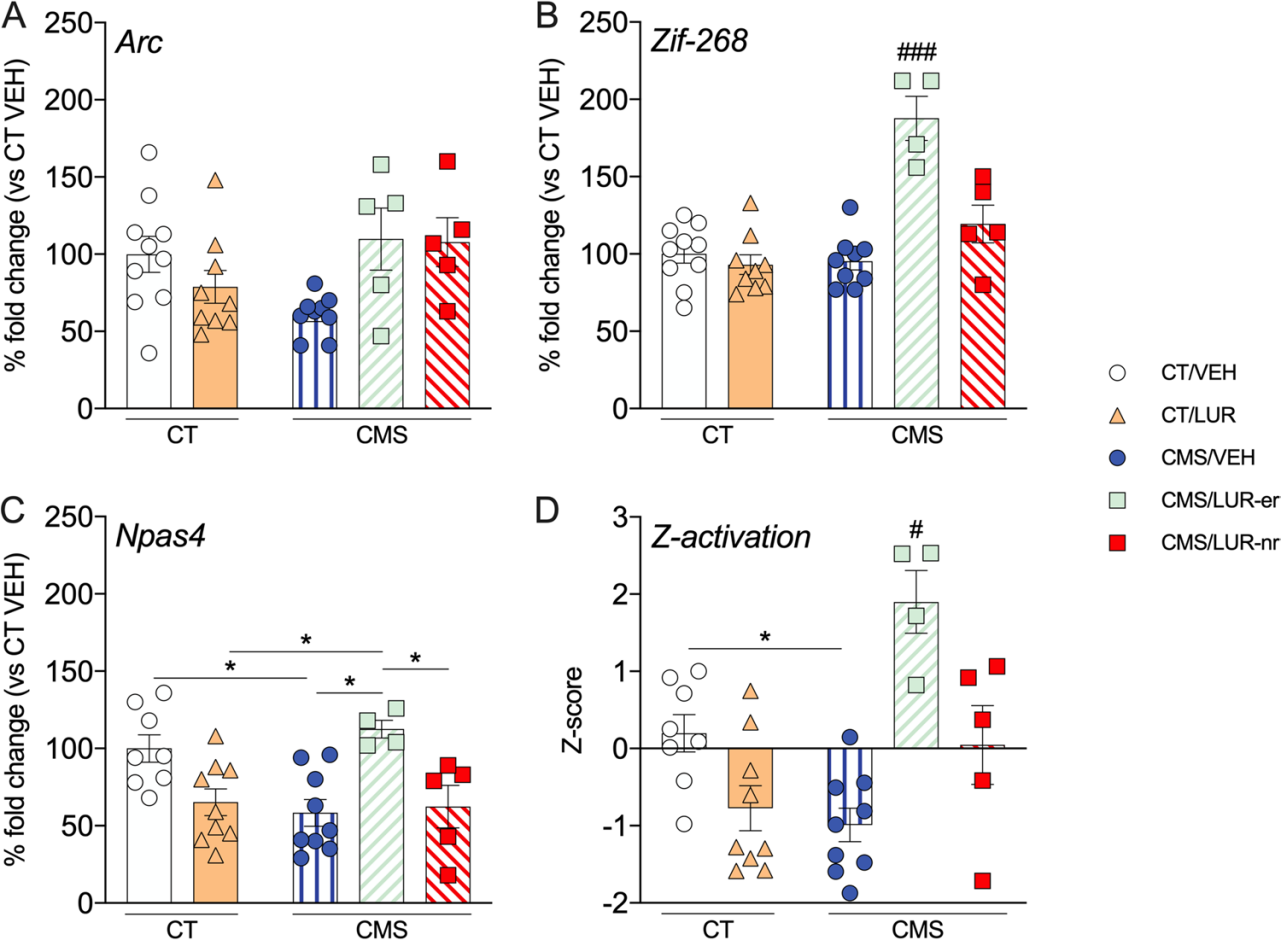
Analysis of the mRNA levels for activity-regulated genes and *Z*-activation score in the prefrontal cortex of CMS rats: modulation by chronic lurasidone treatment. The data shows the mean ± SEM for the mRNA levels of Arc (panel **A**), Zif-268 (panel **B**), Npas4 (panel **C**), as well as the global Z-activation score with 4 to 10 animals per group (panel **D**). The analyses were conducted in control (CT) or stressed (CMS) animals treated with vehicle (VEH) or lurasidone (LUR), discriminating early responders (CMS/LUR-er) from non-responders (CMS/LUR-nr). ^###^*p* < 0.001 vs. every other group, ^#^*p* < 0.05 vs. every other group, *p < 0.05 (one-way ANOVA, Tukey’s *post hoc*)

Within the prefrontal cortex (PFC), we found an overall significant effect on *Arc* mRNA levels (*F* (4, 33) = 3.137, *p* = 0.027, one-way ANOVA), although *post hoc* analysis did not show significant differences between groups (Fig. 2A). A similar overall effect was observed for *Zif-268* mRNA levels (*F* (4, 32) = 17.36, *p* < 0.0001, one-way ANOVA), and *post hoc* analysis revealed that its expression in CMS/LUR-er was significantly different from the other experimental groups (*p* < 0.001; Fig. 2B). Last, the analysis of *Npas4* mRNA levels also revealed an overall significant effect (*F* (4, 30) = 5.681, *p* = 0.0016, one-way ANOVA), whereas Tukey’s *post hoc* test indicated that Npas4 expression in the CMS/LUR-er did not differ from CT/VEH (*p* = 0.927), while it was significantly different from the other groups (*p* < 0.05 for all comparisons; Fig. 2C). Lastly, based on these data, we calculated a *Z*-score to have a better integrated view of the PFC activation and we found an overall significant effect (*F* (4,30) = 10.46, *p* < 0.0001). Indeed, while CMS/VEH rats show a reduction, as compared to CT/VEH, CMS/LUR-er animals show a significant increase in PFC activation when compared to the other experimental groups (*p* < 0.05 for all comparisons; Fig. 2D).

**Fig. 3:**
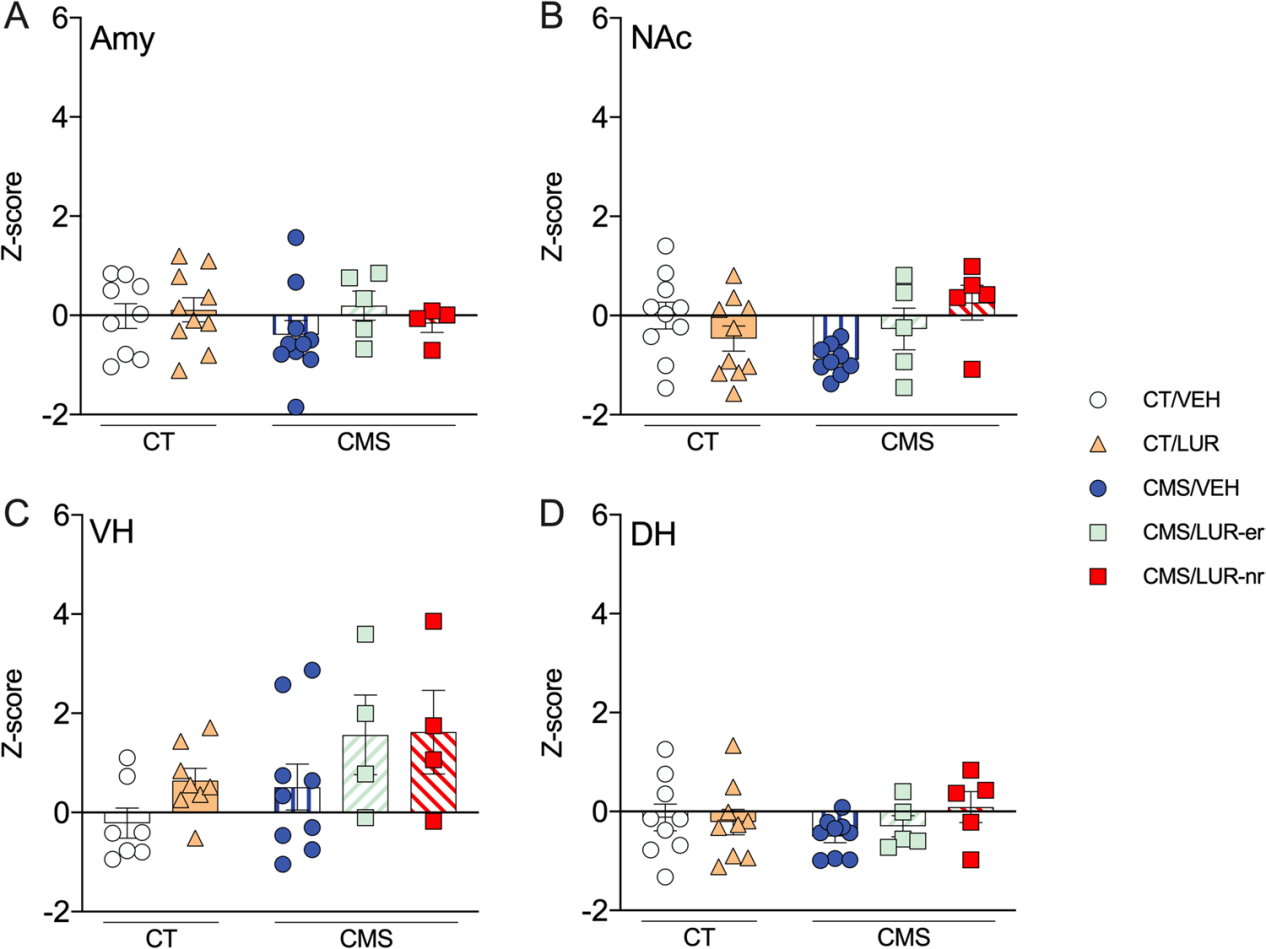
Analysis of global *Z*-activation score for different brain regions of CMS rats: modulation by chronic lurasidone treatment. The data shows mean ± SEM for the global *Z*-activation score of the amygdala (panel **A**), nucleus accumbens (panel **B**), ventral hippocampus (panel **C**), and dorsal hippocampus (panel **D**), with 4 to 10 animals per group. The analyses were conducted in control (CT) or stressed (CMS) animals treated with vehicle (VEH) or lurasidone (LUR), discriminating early responders (CMS/LUR-er) from non-responders (CMS/LUR-nr)

In order to ascertain the region specificity of this mechanism, we also investigated the same activity-dependent genes in other brain areas. With respect to the amygdala, no significant differences were found in the overall analysis. Next, we investigated the individual genes in the nucleus accumbens, where the overall analysis was significant for *Arc* and *Zif-268* (*F* (4,34) = 2.99, *p* = 0.032; *F* (4, 35) = 2.73, *p* = 0.044), but not for *Npas4*. When it comes to the ventral hippocampus, only the overall analysis of *Arc* was significant (*F* (4, 29) = 5.493, *p* = 0.002), whereas no significant changes for any of these genes were found in the DH. Further details of the *post hoc* analysis for these brain regions can be found in Supplementary Figs. S1 and S2. When examining the *Z*-activation score of the abovementioned regions (Fig. 3A–D), no significant differences were found between the different experimental groups (Amy: *p* = 0.56; NAc:* p* = 0.051; VH: *p* = 0.1; DH: *p* = 0.558), suggesting that the PFC may play a key role in the early responsiveness to lurasidone treatment.

### Early lurasidone responders show altered excitatory/inhibitory balance in the PFC

**Fig. 4:**
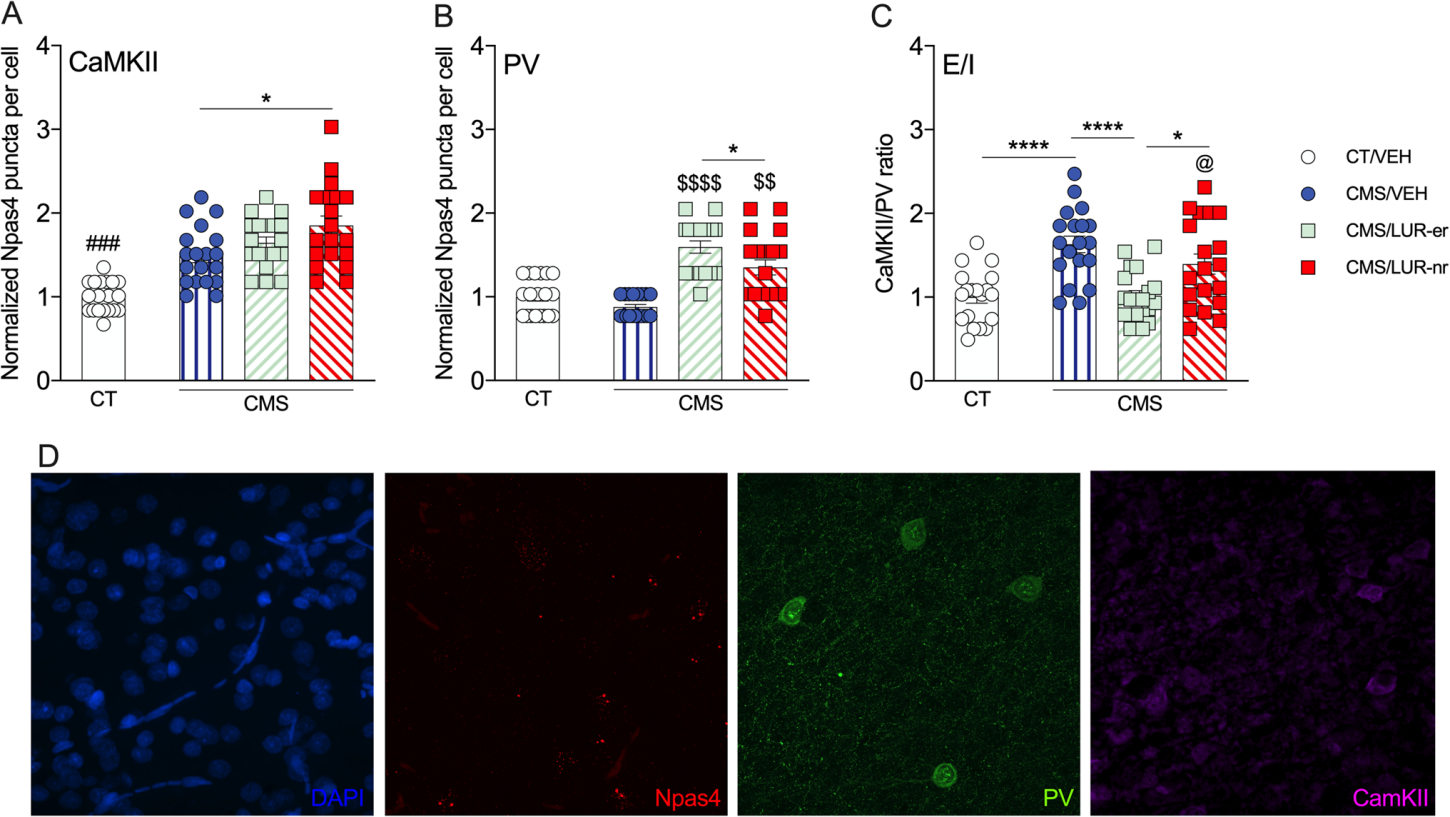
Analysis of Npas4 expression in excitatory and inhibitory neurons of the prefrontal cortex of CMS rats: modulation by chronic lurasidone treatment. The data, based on RNA scope analysis, shows the mean ± SEM for the mRNA levels of Npas4 in CaMKII-positive cells (panel **A**), parvalbumin-positive cells (panel **B**), as well as the ratio of Npas4 expression in excitatory and inhibitory cells (CaMKII/PV) (panel **C**). Representative images of the staining are displayed in panel **D**. The analyses were conducted in control (CT) or stressed (CMS) animals treated with vehicle (VEH) or lurasidone (LUR), discriminating early responders (CMS/LUR-er) from non-responders (CMS/LUR-nr) with* n* = 19 cells for each experimental group. ^###^*p* < 0.001 vs. every other group, ^$$$$^*p* < 0.0001 vs. VEH-treated groups (CT/VEH and CMS/VEH), ^$$^*p* < 0.01 vs. VEH-treated groups (CT/VEH and CMS/VEH), ^@^*p* < 0.05 vs. CT/VEH, ****p < 0.0001, *p < 0.05 (one-way ANOVA, Tukey’s *post hoc*)

Considering that the activation of the prefrontal cortex may contribute to the early responsiveness of lurasidone administration and that the expression pattern of *Npas4* may better reflect the overall activation of this region, we performed RNA scope analysis to investigate Npas4 expression in glutamatergic excitatory pyramidal neurons (CamKII positive) as compared to GABAergic inhibitory neurons (Parvalbumin positive). As already mentioned, Npas4 has an important role in the excitatory/inhibitory (E/I) balance (Fu et al. 2020; Spiegel et al. 2014). We found an overall significant effect of Npas4 in pyramidal neurons (*F* (3, 72) = 20.39, *p* < 0.0001), while post hoc analysis showed that all groups had elevated Npas4 mRNA levels in CamKII-positive cells when compared to the CT/VEH group (*p* < 0.001 for all comparisons; Fig. 4A). Next, we found a significant effect of Npas4 for PV-positive cells (*F* (3, 72) = 25.51, *p* < 0.0001). Tukey’s tests revealed that CMS rats treated with lurasidone (CMS/LUR-er and CMS/LUR-nr) were significantly different from vehicle-treated groups (CT/VEH and CMS/VEH; *p* < 0.01 for all comparisons). Moreover, the analysis also showed increased *Npas4* expression of the CMS/LUR-er group (*p* = 0.04) in PV-positive cells when compared to the CMS/LUR-nr (Fig. 4B).

Last, we calculated the ratio of *Npas4* expression in excitatory and inhibitory neurons (CamKII/PV) to investigate possible alterations in the E/I activation balance. Consistently, the analysis yielded a significant effect (F (3, 72) = 11.14, *p* < 0.0001), and the *post hoc* test revealed that the CMS/VEH and CMS/LUR-nr showed a higher E/I ratio (*p* < 0.05 for both comparisons) when compared to CT/VEH. A similar elevation was observed in CMS/VEH and CMS/LUR-nr when compared to early lurasidone responders (*p* < 0.0001; *p* = 0.023, respectively), whereas no differences were found between CT/VEH and CMS/LUR-er. A representative picture of confocal images is displayed in Fig. 4D.

## Discussion

Previous studies have shown that long-term treatment with antipsychotic or antidepressant drugs is required to normalize the behavioral and functional alterations that may arise as a consequence of stress exposure. As an example, anhedonia, a core domain of different psychiatric disorders, is observed in animals exposed to the chronic mild stress (CMS) paradigm, and such alteration can be normalized upon prolonged exposure to different psychotropic drugs (Orsetti et al. 2007; Papp et al. 2014). Accordingly, we have previously shown that chronic treatment with lurasidone, an antipsychotic drug characterized by a multi-receptor profile, can normalize the anhedonic phenotype in animals exposed to CMS, as well as the stress-induced molecular alterations observed in different brain structures (Begni et al. 2022; Brivio et al. 2021; Calabrese et al. 2020; Calabrese et al. 2016; Luoni et al. 2014). While such alterations were characterized at the end of the chronic treatment, the behavioral effects of lurasidone develop progressively during the course of drug administration. Within this context, in the present study, we show that the anhedonic phenotype was normalized after two weeks of lurasidone in 50% of the animals. Interestingly, we demonstrate that such “early responsiveness” to lurasidone is associated with increased prefrontal cortex activation. Indeed, CMS/LUR-er animals showed increased mRNA levels of different activity-dependent *genes* as well as a significant elevation of the Z-Activation score in the PFC.

Prefrontal cortex tissue from both chronically stressed mice and from clinically depressed human patients display reduced expression of immediate early genes, indicative of decreased activity of this brain region (Covington et al. 2010). The PFC is characterized by efferent and afferent projections to and from several brain regions and plays a key role in different pathologic domains of mental disorders, including the reward system closely associated with anhedonia. Accordingly, abnormal PFC functioning has been linked to anhedonic behavior (Der-Avakian & Markou 2012; Liang et al. 2022). Additionally, a meta-analysis of human neuroimaging studies that evaluated the effects of different antipsychotic drugs on prefrontal activation showed that second-generation antipsychotics may lead to a stronger activation of the PFC when compared to older drugs (Liemburg et al. 2012).

With this respect, microdialysis studies have shown that lurasidone administration is able to increase the release of different neurotransmitters, including dopamine and acetylcholine, in the rat prefrontal cortex, an effect that may depend upon its ability to modulate 5-HT_1a_ and 5-HT_7_ receptors. Furthermore, it has been shown that GABA release in the mPFC is tonically inhibited by 5-HT_7_ receptor stimulation, suggesting that the antagonism at this receptor might be clinically useful to enhance cortical GABAergic release (Huang et al. 2018). Furthermore, chronic lurasidone treatment may enhance serotonergic transmission by desensitizing both 5-HT_1a_ and 5-HT_7_ receptors. We suggest that the ability to modulate different neurotransmitters, particularly in the prefrontal cortex, produces adaptive changes culminating with an increased activity that may contribute to its ability in normalizing CMS-induced anhedonia. With that in mind, we speculate that restoring the expression of activity-regulated genes, and in particular *Npas4*, may represent one possible mechanism underlying the behavioral improvement observed following lurasidone administration.

Npas4 is an activity-dependent gene that is part of homeostatic plasticity mechanisms and has an important role in the regulation of E/I balance, since its altered expression may lead to a balance disruption (Fu et al. 2020; Spiegel et al. 2014). Indeed, using RNA scope, we observed that CMS exposure increases Npas4 mRNA levels in excitatory neurons without affecting its levels in PV-positive cells, which results in an E/I unbalance possibly leading to a PFC overexcitation. Such alteration is not observed in CMS rats treated with lurasidone that showed normalized anhedonic phenotype after 2 weeks of treatment, suggesting that E/I balance restoration in the PFC may represent one of the mechanisms underlying the early responsiveness to the pharmacological intervention. In accordance with our data, an E/I unbalance within the PFC is known to be a consequence of chronic stress and may represent a trait marker of several mental disorders (Bittar & Labonté, 2021; Lee et al. 2017; Liu et al. 2021; Sohal & Rubenstein 2019). Furthermore, male rats exposed to CMS show higher excitability in pyramidal cells of the PFC (Czéh et al. 2018), although it is important to note that there are some conflicting evidence on E/I balance disruption, which may depend upon differences in stress type as well as sex and species (Page & Coutellier 2019).

Anhedonia is a key pathologic domain shared by different psychiatric conditions. When considering schizophrenia, anhedonia is part of the negative symptoms that appear to be more persistent and treatment resistant. Considering that the endurance of negative symptoms is a major cause of patient disability, there is a great need for drugs that can effectively target and ameliorate such symptoms (Correll & Schooler 2020). While it is difficult to translate our results into the clinical setting, it is interesting to mention that depressed patients acutely treated with lurasidone show a normalized anterior cingulate cortex (ACC) activity when compared to placebo during a reward-based task (Wolke et al. 2019). Importantly, the ACC is strongly connected to the PFC and has an important role in decision-making and in reward processes (Apps & Ramnani 2014; Bush et al. 2002; Stevens et al. 2011).

All in all, the results of this study provide further support for the ability of lurasidone treatment in counteracting the alterations produced upon exposure to chronic stress. Moreover, our data suggest that the modulation of the prefrontal cortex represents an important mechanism for the early responsiveness to lurasidone administration and this may promote adaptive changes that boost long-term resilience.

## Supplementary Information

Supplementary file 1 (PDF 173 KB)
