# Supplementary material for: Early effects of lurasidone treatment in a chronic mild stress model in male rats
Source: Psychopharmacology (Berl). 2023 Feb 23;240(4):1001–10. doi: 10.1007/s00213-023-06343-5 (PMC10006266; doi:10.1007/s00213-023-06343-5)
Supplement: Supplementary file 1 — (PDF 173 KB) [file 213_2023_6343_MOESM1_ESM.pdf]

**Psychopharmacology**

# Early effects of Lurasidone treatment in a chronic mild stress model in male rats


Kerstin Camile Creutzberg[a], Veronica Begni[a], Francesca Marchisella[a], Mariusz Papp[c], and Marco Andrea Riva[a,b]

[a]Department of Pharmacological and Biomolecular Sciences, University of Milan, Milan, Italy.
[b]Biological Psychiatry Unit, IRCCS Istituto Centro San Giovanni di Dio Fatebenefratelli, Brescia, Italy.
[c]Maj Institute of Pharmacology, Polish Academy of Sciences, Krakow, Poland.

*Full postal addresses:*
[a]Department of Pharmacological and Biomolecular Sciences, University of Milan – Via Balzaretti 9, 20133 Milan (Italy)
[b]Biological Psychiatry Unit, IRCCS Istituto Centro San Giovanni di Dio Fatebenefratelli – Via Pilastroni 4, 25125 Brescia (Italy)
[c]Maj Institute of Pharmacology, Polish Academy of Sciences – Smętna 12, 31-343 Krakow (Poland)

*Communicating author:*
Marco Andrea Riva
e-mail address: m.riva@unimi.it

# Supplementary material

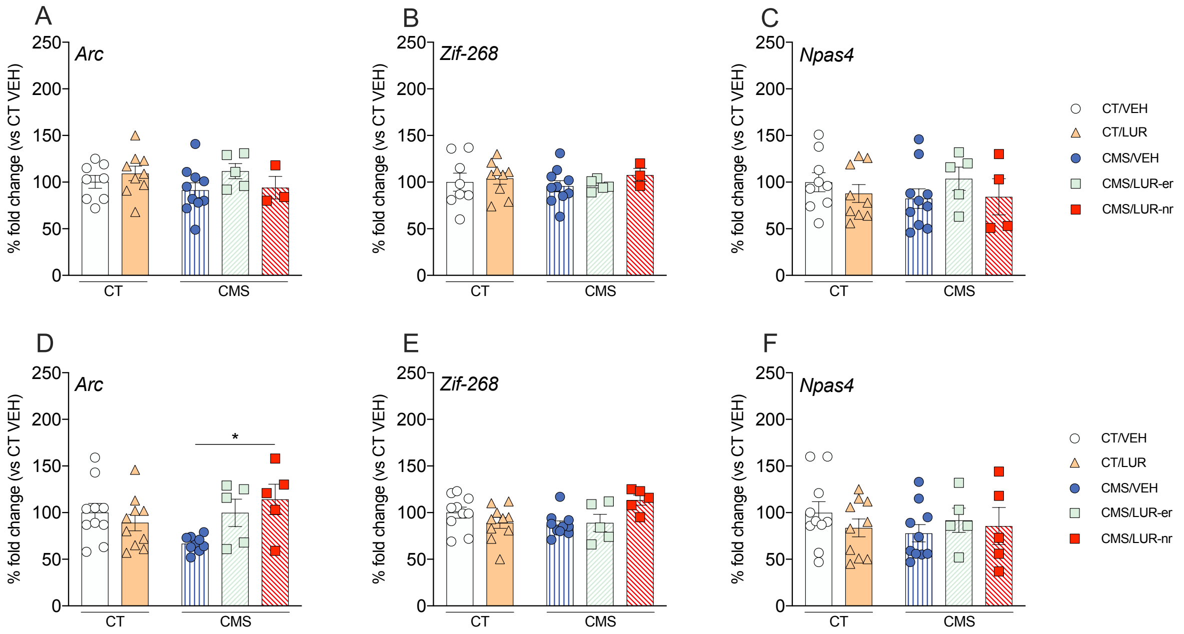


**Figure S1. Analysis of the mRNA levels for activity-regulated genes in the amygdala and nucleus accumbens of CMS rats: modulation by chronic lurasidone treatment.**

The data show the mean ± SEM for the mRNA levels of Arc (panel A), Zif-268 (panel B), and Npas4 (panel C) in the amygdala, as well of Arc (panel D), Zif-268 (panel E) and Npas4 (panel F) in the nucleus accumbens, with 3 to 10 animals per group. The analyses were conducted in control (CT) or stressed (CMS) animals treated with vehicle (VEH) or lurasidone (LUR), discriminating early responders (CMS/LUR-er) from non-responders (CMS/LUR-nr). *p < 0.05 (One-way ANOVA, Tukey's post hoc).

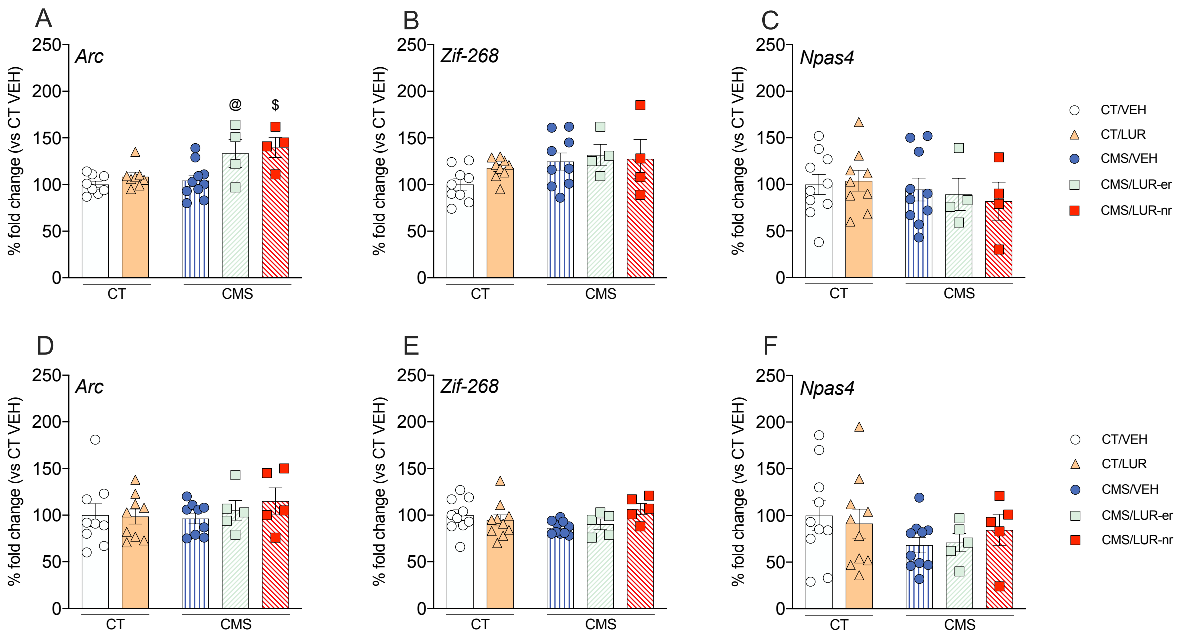


**Figure S2. Analysis of the mRNA levels for activity-regulated genes in the ventral and dorsal hippocampus of CMS rats: modulation by chronic lurasidone treatment.**

The data show the mean ± SEM for the mRNA levels of Arc (panel A), Zif-268 (panel B), and Npas4 (panel C) in the ventral hippocampus, as well of Arc (panel D), Zif-268 (panel E) and Npas4 (panel F) in the dorsal hippocampus, with 4 to 10 animals per group. The analyses were conducted in control (CT) or stressed (CMS) animals treated with vehicle (VEH) or lurasidone (LUR), discriminating early responders (CMS/LUR-er) from non-responders (CMS/LUR-nr). $^{@}p < 0.05$ vs CT/VEH, $^{\$}p < 0.05$ vs VEH treated groups (CT/VEH and CMS/VEH) (One-way ANOVA, Tukey's *post hoc*).
